# Bis[1,2-bis­(diphenyl­phosphino)ethane-κ^2^ *P*:*P*′]silver(I) bis­(chloro­difluoro­acetato-κ*O*)(4-chloro­phen­yl)diphenyl­stannate(IV)

**DOI:** 10.1107/S1600536808014128

**Published:** 2008-05-17

**Authors:** Yin Yin Teo, Kong Mun Lo, Seik Weng Ng

**Affiliations:** a Department of Chemistry, University of Malaya, 50603 Kuala Lumpur, Malaysia

## Abstract

In the title salt, [Ag(C_26_H_24_P_2_)_2_][Sn(C_2_ClF_2_O_2_)_2_(C_6_H_5_)_2_(C_6_H_4_Cl)], the Ag^I^ atom has a tetra­hedral and the Sn^IV^ atom a *trans*-trigonal-bipyramidal coordination geometry. In the anion, the chloro substituent is disordered over two rings (occupancy ratio 0.81:0.19); the two chloro­difluoro­methyl groups are also disordered over two sites for their halogen atoms (occupancy ratios 0.72:0.28 and 0.70:0.30).

## Related literature

For other [1,2-bis­(diphenyl­phosphino)ethane]silver bis­(chloro­­difluoro­acetato)triorganostannates, see: Teo *et al.* (2007 ▶; 2008 ▶). The structural chemistry of organotin carboxyl­ates has been reviewed by Tiekink (1991 ▶, 1994 ▶).
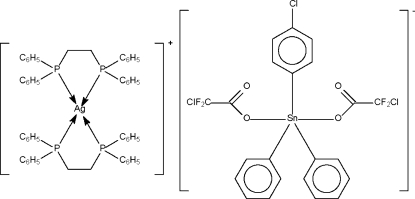


## Experimental

### 

#### Crystal data

[Ag(C_26_H_24_P_2_)_2_][Sn(C_2_ClF_2_O_2_)_2_(C_6_H_5_)_2_(C_6_H_4_Cl)]
*M* *_r_* = 1548.03
Triclinic, 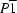

*a* = 13.4774 (3) Å
*b* = 15.4957 (5) Å
*c* = 18.1475 (4) Å
α = 69.674 (2)°
β = 84.646 (2)°
γ = 74.030 (2)°
*V* = 3416.8 (2) Å^3^
*Z* = 2
Mo *K*α radiation
μ = 0.92 mm^−1^
*T* = 100 (2) K
0.19 × 0.09 × 0.06 mm

#### Data collection

Bruker SMART APEX diffractometer
Absorption correction: multi-scan *SADABS* (Sheldrick, 1996 ▶) *T*_min_ = 0.844, *T*_max_ = 0.947
37182 measured reflections
16111 independent reflections
10301 reflections with *I* > 2σ(*I*)
*R*_int_ = 0.075

#### Refinement

*R*[*F* ^2^ > 2σ(*F* ^2^)] = 0.057
*wR*(*F* ^2^) = 0.148
*S* = 1.01
16111 reflections
868 parameters
118 restraints
H-atom parameters constrained
Δρ_max_ = 1.35 e Å^−3^
Δρ_min_ = −1.76 e Å^−3^

### 

Data collection: *APEX2* (Bruker, 2007 ▶); cell refinement: *SAINT* (Bruker, 2007 ▶); data reduction: *SAINT*; program(s) used to solve structure: *SHELXS97* (Sheldrick, 2008 ▶); program(s) used to refine structure: *SHELXL97* (Sheldrick, 2008 ▶); molecular graphics: *X-SEED* (Barbour, 2001 ▶); software used to prepare material for publication: *publCIF* (Westrip, 2008 ▶).

## Supplementary Material

Crystal structure: contains datablocks global, I. DOI: 10.1107/S1600536808014128/bq2076sup1.cif

Structure factors: contains datablocks I. DOI: 10.1107/S1600536808014128/bq2076Isup2.hkl

Additional supplementary materials:  crystallographic information; 3D view; checkCIF report

## Figures and Tables

**Table 1:** Selected bond angles (°)

C1—Sn1—C7	127.5 (2)
C1—Sn1—C13	112.4 (2)
C1—Sn1—O1	85.5 (2)
C7—Sn1—O1	91.8 (2)
C7—Sn1—C13	120.1 (2)
C7—Sn1—O3	89.9 (2)
O1—Sn1—O3	176.6 (1)
P1—Ag1—P3	132.80 (5)
P2—Ag1—P4	119.90 (4)
P3—Ag1—P4	83.87 (5)

**Fig. 1.**
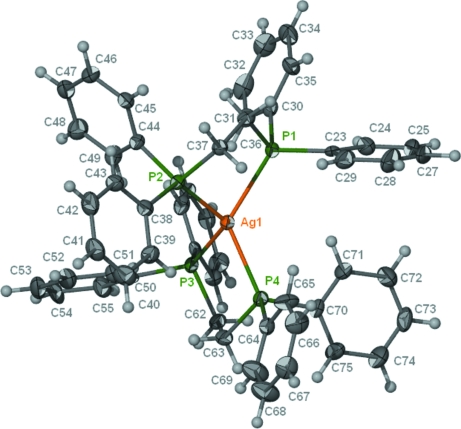
70% Probability thermal ellipsoid plot (Barbour, 2001) of the [Ag(C26H24P2)2] cation. Hydrogen atoms are drawn as spheres of arbitrary radii.

**Fig. 2.**
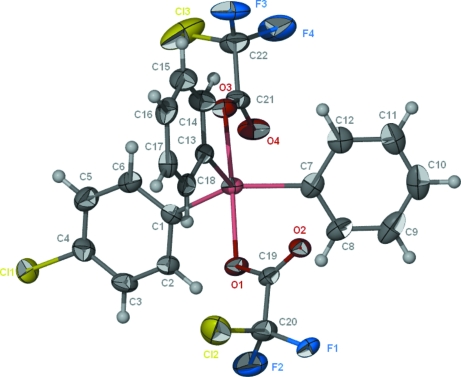
70% Probability thermal ellipsoid plot (Barbour, 2001) of the [Sn(ClC6H4)(C6H5)2(C2ClF2O2)] anion. The disordered atoms are not shown. Hydrogen atoms are drawn as spheres of arbitrary radii.

## supplementary crystallographic information

### Comment

This study continues with studies on bis[1,2-bis(diphenylphosphine)ethane]silver bis(chlorodifluoroacetato)triorganostannates (Teo *et al.*, 2007, 2008). In the present study, the [Ag(Ph_2_CH_2_CH_2_Ph_2_)_2_] cation is the counterion for [Sn(C_6_H_4_Cl)Ph_2_(ClF_2_CCO_2_)_2_] (Scheme I). The silver(I) atom shows tetrahedral and the tin atom *trans*-trigonal bipyramidal coordination (Fig. 1).

### Experimental

(4-Chlorophenyl)diphenyltin hydroxide (0.20 g, 0.5 mmol) and chorodifluoroacetic acid (0.05 ml, 0.5 mmol) were dissolved in dichloromethane/methanol (25 ml). The mixture was heated until the hydroxide dissolved completely. Another solution containing 1,2-bis(diphenylphosphino)ethane (0.40 g, 1.0 mmol) and silver trifluoroacetate (0.11 g, 0.5 mmol) was prepared; this was also heated until the reagents dissolved completely. The two solutions were mixed; crystals were obtained by allowing the solvent to evaporate in about 70% yield.

### Refinement

Carbon-bound H-atoms were placed in calculated positions (C—H 0.95 to 0.99 Å) and were included in the refinement in the riding model approximation, with *U*(H) set to 1.2*U*_eq_(C).The chlorine atom of the phenylene ring is disordered over two rings; the C–Cl distances were restrained to 1.75±0.01 Å. The disorder refined to a 0.81:0.19 ratio. The two chlorodifluoromethyl groups of the chlorodifluoroacetate anion are both disordered. Distance restraints were applied: C–C distances = 1.50±0.01 Å, C–Cl 1.75±0.01 Å; C–F 1.35±0.01 Å; Cl···F 2.52±0.01 Å and F···F 2.21±0.01 Å. Additionally, the six halogen atoms of each anion were restrained to lie in an approximate plane, the atoms being allowed to deviate by a maximum of 0.05 Å. The anisotropic temperature factors of the disordered atoms were restrained to be nearly isotropic. The final difference Fourier map had a large peak/hole (-1.76 e Å^3^ in the vicinity of one of the two disordered anions.

### Crystal data

**Table:**

[Ag(C_26_H_24_P_2_)_2_][Sn(C_2_ClF_2_O_2_)_2_(C_6_H_5_)_2_(C_6_H_4_Cl)]	*Z* = 2
*M**_r_* = 1548.03	*F*_000_ = 1564
Triclinic, *P*1	*D*_x_ = 1.505 Mg m^−^^3^
Hall symbol: -P 1	Mo *K*α radiation λ = 0.71073 Å
*a* = 13.4774 (3) Å	Cell parameters from 3393 reflections
*b* = 15.4957 (5) Å	θ = 2.3–21.7º
*c* = 18.1475 (4) Å	µ = 0.92 mm^−^^1^
α = 69.674 (2)º	*T* = 100 (2) K
β = 84.646 (2)º	Block, colorless
γ = 74.030 (2)º	0.19 × 0.09 × 0.06 mm
*V* = 3416.8 (2) Å^3^	

### Data collection

**Table:**

Bruker SMART APEX diffractometer	16111 independent reflections
Radiation source: fine-focus sealed tube	10301 reflections with *I* > 2σ(*I*)
Monochromator: graphite	*R*_int_ = 0.076
*T* = 100(2) K	θ_max_ = 27.5º
ω scans	θ_min_ = 1.2º
Absorption correction: Multi-scanSADABS (Sheldrick, 1996)	*h* = −17→17
*T*_min_ = 0.844, *T*_max_ = 0.947	*k* = −20→15
37182 measured reflections	*l* = −23→23

### Refinement

**Table:**

Refinement on *F*^2^	Secondary atom site location: difference Fourier map
Least-squares matrix: full	Hydrogen site location: inferred from neighbouring sites
*R*[*F*^2^ > 2σ(*F*^2^)] = 0.057	H-atom parameters constrained
*wR*(*F*^2^) = 0.148	*w* = 1/[σ^2^(*F*_o_^2^) + (0.0516*P*)^2^ + 3.4764*P*] where *P* = (*F*_o_^2^ + 2*F*_c_^2^)/3
*S* = 1.01	(Δ/σ)_max_ = 0.001
16111 reflections	Δρ_max_ = 1.36 e Å^−^^3^
868 parameters	Δρ_min_ = −1.76 e Å^−^^3^
118 restraints	Extinction correction: none
Primary atom site location: structure-invariant direct methods	

### Special details

**Table:**

Geometry. All e.s.d.'s (except the e.s.d. in the dihedral angle between two l.s. planes) are estimated using the full covariance matrix. The cell e.s.d.'s are taken into account individually in the estimation of e.s.d.'s in distances, angles and torsion angles; correlations between e.s.d.'s in cell parameters are only used when they are defined by crystal symmetry. An approximate (isotropic) treatment of cell e.s.d.'s is used for estimating e.s.d.'s involving l.s. planes.

### Fractional atomic coordinates and isotropic or equivalent isotropic displacement parameters (Å^2^)

**Table:**

	*x*	*y*	*z*	*U*_iso_*/*U*_eq_	Occ. (<1)
Sn1	0.18356 (3)	0.41136 (3)	0.74160 (2)	0.02029 (10)	
Ag1	0.34144 (3)	−0.12991 (3)	0.74626 (2)	0.01634 (10)	
Cl1	−0.16052 (14)	0.15843 (14)	0.80533 (11)	0.0334 (5)	0.813 (4)
Cl2	0.13197 (16)	0.43648 (19)	1.02140 (12)	0.0509 (7)	0.813 (4)
Cl3	0.1026 (3)	0.4097 (2)	0.45258 (17)	0.0712 (12)	0.722 (5)
F1	0.3254 (3)	0.3662 (3)	1.02756 (19)	0.0261 (11)	0.813 (4)
F2	0.2350 (4)	0.2721 (3)	1.0236 (2)	0.0530 (14)	0.813 (4)
F3	0.1652 (5)	0.5441 (3)	0.4608 (3)	0.0505 (17)	0.722 (5)
F4	0.2835 (4)	0.4236 (5)	0.4476 (3)	0.077 (3)	0.722 (5)
Cl2'	0.2959 (8)	0.2388 (5)	1.0223 (4)	0.0530 (14)	0.19
Cl3'	0.2789 (5)	0.5165 (6)	0.4514 (3)	0.059 (3)	0.278 (5)
F1'	0.2994 (11)	0.3969 (9)	1.0274 (5)	0.0261 (11)	0.19
F2'	0.1466 (7)	0.3712 (11)	1.0226 (5)	0.0509 (7)	0.19
F3'	0.0957 (6)	0.5099 (9)	0.4631 (5)	0.074 (5)	0.278 (5)
F4'	0.2082 (12)	0.3847 (7)	0.4476 (5)	0.071 (5)	0.278 (5)
P1	0.43266 (10)	−0.27742 (10)	0.71408 (7)	0.0162 (3)	
P2	0.26737 (10)	−0.24426 (10)	0.85526 (7)	0.0157 (3)	
P3	0.40448 (10)	−0.00758 (10)	0.77467 (7)	0.0170 (3)	
P4	0.22324 (10)	0.01273 (10)	0.65085 (7)	0.0170 (3)	
O1	0.1970 (3)	0.3585 (3)	0.8728 (2)	0.0250 (9)	
O2	0.2980 (3)	0.4534 (3)	0.8737 (2)	0.0236 (8)	
O3	0.1653 (3)	0.4574 (3)	0.6122 (2)	0.0249 (9)	
O4	0.2539 (3)	0.3173 (3)	0.5987 (2)	0.0342 (10)	
C1	0.0862 (4)	0.3184 (4)	0.7596 (3)	0.0210 (12)	
C2	0.0951 (4)	0.2341 (4)	0.8233 (3)	0.0231 (12)	
H2	0.1525	0.2116	0.8579	0.028*	
C3	0.0214 (5)	0.1825 (4)	0.8372 (3)	0.0287 (13)	
H3	0.0291	0.1244	0.8800	0.034*	
C4	−0.0633 (4)	0.2173 (4)	0.7876 (3)	0.0290 (13)	
H4	−0.1156	0.1841	0.7980	0.035*	0.187 (4)
C5	−0.0729 (5)	0.2994 (5)	0.7231 (4)	0.0351 (15)	
H5	−0.1299	0.3213	0.6883	0.042*	
C6	0.0012 (4)	0.3493 (4)	0.7100 (3)	0.0298 (14)	
H6	−0.0059	0.4062	0.6660	0.036*	
C7	0.3472 (4)	0.3784 (4)	0.7282 (3)	0.0260 (13)	
C8	0.4138 (5)	0.3012 (4)	0.7814 (3)	0.0297 (14)	
H8	0.3861	0.2587	0.8242	0.036*	
C9	0.5190 (5)	0.2854 (5)	0.7730 (4)	0.0380 (17)	
H9	0.5637	0.2312	0.8085	0.046*	
C10	0.5589 (5)	0.3497 (5)	0.7120 (4)	0.0391 (17)	
H10	0.6316	0.3407	0.7076	0.047*	0.813 (4)
Cl1'	0.6905 (9)	0.3299 (19)	0.7021 (15)	0.171 (10)	0.187 (4)
C11	0.4963 (5)	0.4253 (5)	0.6585 (4)	0.0346 (15)	
H11	0.5251	0.4674	0.6160	0.042*	
C12	0.3899 (5)	0.4407 (5)	0.6663 (3)	0.0304 (14)	
H12	0.3459	0.4939	0.6293	0.036*	
C13	0.0986 (4)	0.5528 (4)	0.7339 (3)	0.0202 (11)	
C14	0.0815 (5)	0.6263 (4)	0.6624 (3)	0.0287 (13)	
H14	0.1089	0.6138	0.6159	0.034*	
C15	0.0251 (5)	0.7179 (4)	0.6573 (4)	0.0337 (15)	
H15	0.0141	0.7669	0.6075	0.040*	
C16	−0.0152 (4)	0.7388 (4)	0.7241 (3)	0.0313 (14)	
H16	−0.0527	0.8017	0.7206	0.038*	
C17	0.0001 (4)	0.6661 (4)	0.7957 (3)	0.0274 (13)	
H17	−0.0277	0.6790	0.8420	0.033*	
C18	0.0561 (4)	0.5738 (4)	0.8008 (3)	0.0249 (12)	
H18	0.0655	0.5246	0.8506	0.030*	
C19	0.2499 (4)	0.3938 (4)	0.9047 (3)	0.0210 (12)	
C20	0.2436 (4)	0.3606 (4)	0.9940 (3)	0.0322 (14)	
C21	0.2060 (4)	0.4013 (4)	0.5744 (3)	0.0254 (13)	
C22	0.1933 (4)	0.4485 (4)	0.4850 (3)	0.0371 (16)	
C23	0.4185 (4)	−0.2828 (4)	0.6172 (3)	0.0194 (11)	
C24	0.4972 (4)	−0.2732 (4)	0.5614 (3)	0.0277 (13)	
H24	0.5598	−0.2644	0.5738	0.033*	
C25	0.4830 (6)	−0.2766 (5)	0.4875 (3)	0.0388 (17)	
H25	0.5367	−0.2709	0.4497	0.047*	
C27	0.3918 (6)	−0.2882 (5)	0.4686 (3)	0.045 (2)	
H27	0.3831	−0.2907	0.4181	0.054*	
C28	0.3134 (5)	−0.2961 (5)	0.5227 (3)	0.0406 (17)	
H28	0.2507	−0.3042	0.5095	0.049*	
C29	0.3258 (5)	−0.2922 (4)	0.5963 (3)	0.0286 (13)	
H29	0.2707	−0.2960	0.6330	0.034*	
C30	0.5692 (4)	−0.3352 (4)	0.7361 (3)	0.0180 (11)	
C31	0.6268 (4)	−0.2935 (4)	0.7664 (3)	0.0205 (11)	
H31	0.5951	−0.2347	0.7745	0.025*	
C32	0.7299 (4)	−0.3368 (5)	0.7848 (3)	0.0300 (14)	
H32	0.7692	−0.3073	0.8048	0.036*	
C33	0.7756 (4)	−0.4231 (5)	0.7742 (3)	0.0338 (15)	
H33	0.8464	−0.4530	0.7870	0.041*	
C34	0.7198 (4)	−0.4656 (5)	0.7454 (3)	0.0327 (15)	
H34	0.7518	−0.5250	0.7383	0.039*	
C35	0.6161 (4)	−0.4223 (4)	0.7264 (3)	0.0234 (12)	
H35	0.5773	−0.4523	0.7067	0.028*	
C36	0.3707 (4)	−0.3694 (4)	0.7792 (3)	0.0158 (10)	
H36A	0.3654	−0.4119	0.7506	0.019*	
H36B	0.4160	−0.4086	0.8253	0.019*	
C37	0.2626 (4)	−0.3305 (4)	0.8087 (3)	0.0178 (11)	
H37A	0.2381	−0.3836	0.8471	0.021*	
H37B	0.2135	−0.2994	0.7641	0.021*	
C38	0.1386 (4)	−0.2200 (4)	0.8980 (3)	0.0187 (11)	
C39	0.0693 (4)	−0.1315 (4)	0.8647 (3)	0.0233 (12)	
H39	0.0888	−0.0856	0.8197	0.028*	
C40	−0.0283 (4)	−0.1102 (5)	0.8972 (3)	0.0309 (14)	
H40	−0.0753	−0.0497	0.8747	0.037*	
C41	−0.0561 (4)	−0.1777 (5)	0.9623 (3)	0.0295 (14)	
H41	−0.1228	−0.1634	0.9843	0.035*	
C42	0.0110 (4)	−0.2650 (5)	0.9956 (3)	0.0314 (14)	
H42	−0.0093	−0.3109	1.0403	0.038*	
C43	0.1082 (4)	−0.2862 (4)	0.9639 (3)	0.0246 (13)	
H43	0.1548	−0.3467	0.9874	0.030*	
C44	0.3491 (4)	−0.3121 (4)	0.9418 (3)	0.0193 (11)	
C45	0.3547 (4)	−0.4080 (4)	0.9852 (3)	0.0197 (11)	
H45	0.3225	−0.4432	0.9658	0.024*	
C46	0.4067 (4)	−0.4511 (4)	1.0557 (3)	0.0254 (13)	
H46	0.4099	−0.5159	1.0850	0.031*	
C47	0.4545 (4)	−0.4003 (4)	1.0844 (3)	0.0273 (13)	
H47	0.4880	−0.4296	1.1341	0.033*	
C48	0.4532 (5)	−0.3073 (4)	1.0405 (3)	0.0301 (14)	
H48	0.4882	−0.2733	1.0590	0.036*	
C49	0.4007 (4)	−0.2631 (4)	0.9691 (3)	0.0209 (12)	
H49	0.4003	−0.1992	0.9390	0.025*	
C50	0.3564 (4)	0.0103 (4)	0.8679 (3)	0.0183 (11)	
C51	0.2711 (4)	−0.0222 (4)	0.9024 (3)	0.0238 (12)	
H51	0.2355	−0.0477	0.8757	0.029*	
C52	0.2381 (5)	−0.0172 (4)	0.9757 (3)	0.0312 (14)	
H52	0.1807	−0.0407	0.9997	0.037*	
C53	0.2886 (5)	0.0219 (5)	1.0147 (3)	0.0371 (17)	
H53	0.2660	0.0252	1.0651	0.044*	
C54	0.3704 (5)	0.0554 (5)	0.9797 (3)	0.0355 (16)	
H54	0.4047	0.0822	1.0061	0.043*	
C55	0.4055 (4)	0.0513 (4)	0.9055 (3)	0.0278 (13)	
H55	0.4620	0.0763	0.8813	0.033*	
C56	0.5389 (4)	−0.0037 (4)	0.7712 (3)	0.0170 (11)	
C57	0.5746 (4)	0.0730 (4)	0.7224 (3)	0.0242 (12)	
H57	0.5268	0.1291	0.6919	0.029*	
C58	0.6785 (4)	0.0689 (4)	0.7176 (3)	0.0268 (13)	
H58	0.7016	0.1223	0.6847	0.032*	
C59	0.7492 (4)	−0.0138 (4)	0.7611 (3)	0.0227 (12)	
H59	0.8211	−0.0179	0.7561	0.027*	
C60	0.7153 (4)	−0.0889 (4)	0.8112 (3)	0.0228 (12)	
H60	0.7639	−0.1439	0.8423	0.027*	
C61	0.6111 (4)	−0.0863 (4)	0.8173 (3)	0.0213 (12)	
H61	0.5885	−0.1392	0.8519	0.026*	
C62	0.3403 (4)	0.1056 (4)	0.7001 (3)	0.0213 (12)	
H62A	0.3406	0.1591	0.7178	0.026*	
H62B	0.3795	0.1132	0.6503	0.026*	
C63	0.2292 (4)	0.1095 (4)	0.6854 (3)	0.0210 (11)	
H63A	0.1973	0.1716	0.6456	0.025*	
H63B	0.1893	0.1046	0.7346	0.025*	
C64	0.0860 (4)	0.0357 (4)	0.6307 (3)	0.0192 (11)	
C65	0.0534 (4)	−0.0335 (4)	0.6162 (3)	0.0298 (14)	
H65	0.1001	−0.0941	0.6228	0.036*	
C66	−0.0472 (5)	−0.0158 (5)	0.5919 (3)	0.0333 (14)	
H66	−0.0682	−0.0636	0.5807	0.040*	
C67	−0.1161 (4)	0.0700 (5)	0.5841 (3)	0.0304 (14)	
H67	−0.1845	0.0827	0.5665	0.037*	
C68	−0.0852 (5)	0.1373 (5)	0.6019 (4)	0.0394 (16)	
H68	−0.1335	0.1963	0.5983	0.047*	
C69	0.0162 (5)	0.1213 (5)	0.6254 (4)	0.0375 (16)	
H69	0.0365	0.1689	0.6374	0.045*	
C70	0.2744 (4)	0.0472 (4)	0.5509 (3)	0.0189 (11)	
C71	0.3469 (5)	−0.0209 (4)	0.5279 (3)	0.0289 (14)	
H71	0.3717	−0.0831	0.5647	0.035*	
C72	0.3835 (5)	0.0010 (5)	0.4515 (3)	0.0395 (17)	
H72	0.4336	−0.0461	0.4360	0.047*	
C73	0.3481 (5)	0.0902 (5)	0.3983 (3)	0.0346 (15)	
H73	0.3729	0.1048	0.3458	0.042*	
C74	0.2759 (5)	0.1593 (5)	0.4209 (3)	0.0384 (16)	
H74	0.2520	0.2217	0.3842	0.046*	
C75	0.2390 (5)	0.1371 (4)	0.4968 (3)	0.0324 (15)	
H75	0.1887	0.1842	0.5121	0.039*	

### Atomic displacement parameters (Å^2^)

**Table:**

	*U*^11^	*U*^22^	*U*^33^	*U*^12^	*U*^13^	*U*^23^
Sn1	0.0262 (2)	0.0174 (2)	0.01686 (18)	−0.00631 (16)	−0.00245 (14)	−0.00419 (15)
Ag1	0.0203 (2)	0.0146 (2)	0.01421 (18)	−0.00492 (16)	0.00009 (14)	−0.00477 (15)
Cl1	0.0331 (10)	0.0287 (11)	0.0412 (11)	−0.0200 (9)	−0.0059 (8)	−0.0043 (8)
Cl2	0.0482 (13)	0.0701 (18)	0.0350 (11)	−0.0151 (12)	0.0073 (9)	−0.0204 (11)
Cl3	0.100 (3)	0.0479 (19)	0.0606 (18)	−0.0052 (17)	−0.0476 (17)	−0.0114 (14)
F1	0.028 (2)	0.028 (3)	0.0202 (16)	−0.005 (2)	−0.0082 (15)	−0.0055 (16)
F2	0.066 (4)	0.043 (3)	0.043 (2)	−0.018 (3)	−0.014 (3)	0.001 (2)
F3	0.086 (4)	0.035 (3)	0.025 (3)	−0.022 (3)	−0.011 (3)	0.004 (2)
F4	0.059 (4)	0.111 (6)	0.024 (3)	0.016 (4)	0.010 (3)	−0.009 (3)
Cl2'	0.066 (4)	0.043 (3)	0.043 (2)	−0.018 (3)	−0.014 (3)	0.001 (2)
Cl3'	0.066 (5)	0.073 (5)	0.037 (3)	−0.033 (4)	0.015 (3)	−0.010 (3)
F1'	0.028 (2)	0.028 (3)	0.0202 (16)	−0.005 (2)	−0.0082 (15)	−0.0055 (16)
F2'	0.0482 (13)	0.0701 (18)	0.0350 (11)	−0.0151 (12)	0.0073 (9)	−0.0204 (11)
F3'	0.077 (9)	0.089 (10)	0.045 (7)	−0.018 (8)	−0.024 (7)	−0.004 (7)
F4'	0.096 (10)	0.076 (9)	0.058 (8)	−0.036 (8)	−0.007 (7)	−0.030 (7)
P1	0.0172 (7)	0.0170 (7)	0.0140 (6)	−0.0032 (6)	−0.0009 (5)	−0.0056 (5)
P2	0.0164 (6)	0.0152 (7)	0.0145 (6)	−0.0049 (5)	0.0002 (5)	−0.0033 (5)
P3	0.0220 (7)	0.0161 (7)	0.0131 (6)	−0.0056 (6)	−0.0007 (5)	−0.0046 (5)
P4	0.0192 (7)	0.0164 (7)	0.0156 (6)	−0.0049 (6)	−0.0015 (5)	−0.0049 (5)
O1	0.033 (2)	0.028 (2)	0.0170 (18)	−0.0128 (19)	−0.0060 (15)	−0.0059 (16)
O2	0.028 (2)	0.019 (2)	0.0230 (19)	−0.0091 (17)	−0.0015 (16)	−0.0025 (16)
O3	0.032 (2)	0.024 (2)	0.0184 (18)	−0.0064 (18)	−0.0020 (16)	−0.0073 (17)
O4	0.053 (3)	0.017 (2)	0.029 (2)	−0.004 (2)	−0.0024 (19)	−0.0054 (18)
C1	0.026 (3)	0.021 (3)	0.019 (3)	−0.009 (2)	0.002 (2)	−0.009 (2)
C2	0.027 (3)	0.025 (3)	0.017 (3)	−0.007 (3)	0.000 (2)	−0.008 (2)
C3	0.040 (4)	0.025 (3)	0.022 (3)	−0.017 (3)	−0.002 (2)	−0.002 (2)
C4	0.032 (3)	0.031 (4)	0.030 (3)	−0.017 (3)	0.000 (2)	−0.010 (3)
C5	0.035 (3)	0.035 (4)	0.035 (3)	−0.012 (3)	−0.009 (3)	−0.007 (3)
C6	0.030 (3)	0.029 (4)	0.027 (3)	−0.008 (3)	−0.004 (2)	−0.004 (3)
C7	0.029 (3)	0.026 (3)	0.028 (3)	−0.005 (3)	−0.005 (2)	−0.017 (3)
C8	0.034 (3)	0.026 (3)	0.028 (3)	−0.002 (3)	−0.007 (2)	−0.011 (3)
C9	0.033 (4)	0.041 (4)	0.046 (4)	0.009 (3)	−0.014 (3)	−0.032 (3)
C10	0.030 (3)	0.060 (5)	0.047 (4)	−0.014 (3)	0.008 (3)	−0.041 (4)
Cl1'	0.168 (13)	0.177 (13)	0.183 (12)	−0.050 (9)	−0.007 (9)	−0.074 (9)
C11	0.036 (4)	0.048 (4)	0.039 (4)	−0.021 (3)	0.008 (3)	−0.032 (3)
C12	0.034 (3)	0.029 (4)	0.034 (3)	−0.008 (3)	0.002 (3)	−0.019 (3)
C13	0.020 (3)	0.023 (3)	0.020 (3)	−0.009 (2)	−0.001 (2)	−0.007 (2)
C14	0.037 (3)	0.023 (3)	0.025 (3)	−0.006 (3)	0.002 (2)	−0.008 (2)
C15	0.039 (4)	0.019 (3)	0.036 (3)	−0.006 (3)	−0.004 (3)	−0.003 (3)
C16	0.024 (3)	0.023 (3)	0.044 (4)	0.001 (3)	−0.004 (3)	−0.012 (3)
C17	0.019 (3)	0.031 (4)	0.038 (3)	−0.005 (3)	0.002 (2)	−0.019 (3)
C18	0.018 (3)	0.030 (3)	0.027 (3)	−0.007 (3)	−0.002 (2)	−0.010 (3)
C19	0.021 (3)	0.022 (3)	0.018 (3)	−0.002 (2)	−0.004 (2)	−0.006 (2)
C20	0.043 (4)	0.034 (4)	0.024 (3)	−0.021 (3)	−0.009 (3)	−0.004 (3)
C21	0.035 (3)	0.028 (3)	0.017 (3)	−0.016 (3)	0.000 (2)	−0.006 (2)
C22	0.044 (4)	0.038 (4)	0.021 (3)	−0.002 (3)	−0.001 (3)	−0.007 (3)
C23	0.026 (3)	0.012 (3)	0.015 (2)	0.005 (2)	−0.004 (2)	−0.003 (2)
C24	0.030 (3)	0.025 (3)	0.024 (3)	−0.001 (3)	0.005 (2)	−0.008 (2)
C25	0.060 (5)	0.026 (4)	0.015 (3)	0.007 (3)	0.004 (3)	−0.004 (3)
C27	0.083 (6)	0.026 (4)	0.019 (3)	0.010 (4)	−0.020 (3)	−0.011 (3)
C28	0.054 (4)	0.040 (4)	0.026 (3)	−0.006 (3)	−0.018 (3)	−0.009 (3)
C29	0.032 (3)	0.026 (3)	0.027 (3)	−0.005 (3)	−0.008 (2)	−0.008 (3)
C30	0.017 (3)	0.022 (3)	0.014 (2)	−0.006 (2)	0.0019 (19)	−0.004 (2)
C31	0.018 (3)	0.022 (3)	0.022 (3)	−0.007 (2)	−0.002 (2)	−0.007 (2)
C32	0.023 (3)	0.045 (4)	0.023 (3)	−0.017 (3)	−0.003 (2)	−0.006 (3)
C33	0.016 (3)	0.045 (4)	0.032 (3)	−0.002 (3)	0.002 (2)	−0.008 (3)
C34	0.027 (3)	0.037 (4)	0.030 (3)	0.002 (3)	0.002 (2)	−0.016 (3)
C35	0.021 (3)	0.026 (3)	0.026 (3)	−0.001 (2)	−0.004 (2)	−0.015 (2)
C36	0.018 (3)	0.011 (3)	0.019 (2)	−0.005 (2)	−0.0043 (19)	−0.003 (2)
C37	0.019 (3)	0.017 (3)	0.017 (2)	−0.004 (2)	−0.003 (2)	−0.006 (2)
C38	0.017 (3)	0.020 (3)	0.020 (3)	−0.003 (2)	−0.003 (2)	−0.009 (2)
C39	0.018 (3)	0.027 (3)	0.021 (3)	−0.002 (2)	−0.002 (2)	−0.005 (2)
C40	0.022 (3)	0.034 (4)	0.037 (3)	−0.001 (3)	−0.004 (2)	−0.017 (3)
C41	0.019 (3)	0.040 (4)	0.032 (3)	−0.003 (3)	0.004 (2)	−0.020 (3)
C42	0.028 (3)	0.042 (4)	0.024 (3)	−0.008 (3)	0.008 (2)	−0.014 (3)
C43	0.024 (3)	0.022 (3)	0.022 (3)	0.000 (2)	0.004 (2)	−0.006 (2)
C44	0.020 (3)	0.018 (3)	0.015 (2)	−0.003 (2)	0.002 (2)	−0.002 (2)
C45	0.021 (3)	0.018 (3)	0.020 (3)	−0.004 (2)	0.003 (2)	−0.008 (2)
C46	0.033 (3)	0.020 (3)	0.020 (3)	−0.003 (3)	−0.003 (2)	−0.004 (2)
C47	0.028 (3)	0.029 (3)	0.023 (3)	0.001 (3)	−0.004 (2)	−0.012 (3)
C48	0.034 (3)	0.030 (4)	0.027 (3)	−0.008 (3)	−0.007 (2)	−0.010 (3)
C49	0.018 (3)	0.028 (3)	0.020 (3)	−0.009 (2)	−0.002 (2)	−0.009 (2)
C50	0.021 (3)	0.013 (3)	0.019 (2)	0.002 (2)	−0.004 (2)	−0.007 (2)
C51	0.028 (3)	0.024 (3)	0.021 (3)	−0.004 (3)	0.000 (2)	−0.011 (2)
C52	0.033 (3)	0.031 (4)	0.025 (3)	−0.003 (3)	0.007 (2)	−0.010 (3)
C53	0.035 (4)	0.047 (4)	0.021 (3)	0.012 (3)	−0.006 (3)	−0.018 (3)
C54	0.033 (3)	0.048 (4)	0.032 (3)	0.005 (3)	−0.010 (3)	−0.032 (3)
C55	0.028 (3)	0.032 (4)	0.027 (3)	−0.004 (3)	−0.005 (2)	−0.016 (3)
C56	0.024 (3)	0.017 (3)	0.015 (2)	−0.008 (2)	−0.001 (2)	−0.011 (2)
C57	0.032 (3)	0.019 (3)	0.020 (3)	−0.005 (3)	0.001 (2)	−0.006 (2)
C58	0.031 (3)	0.025 (3)	0.027 (3)	−0.014 (3)	0.000 (2)	−0.006 (3)
C59	0.024 (3)	0.029 (3)	0.024 (3)	−0.013 (3)	0.001 (2)	−0.014 (2)
C60	0.031 (3)	0.019 (3)	0.017 (3)	−0.004 (2)	−0.005 (2)	−0.004 (2)
C61	0.027 (3)	0.024 (3)	0.017 (2)	−0.012 (2)	−0.001 (2)	−0.006 (2)
C62	0.029 (3)	0.016 (3)	0.018 (3)	−0.004 (2)	−0.007 (2)	−0.004 (2)
C63	0.025 (3)	0.015 (3)	0.023 (3)	−0.004 (2)	−0.003 (2)	−0.008 (2)
C64	0.019 (3)	0.021 (3)	0.016 (2)	−0.003 (2)	0.000 (2)	−0.006 (2)
C65	0.026 (3)	0.019 (3)	0.039 (3)	0.000 (3)	−0.010 (3)	−0.005 (3)
C66	0.032 (3)	0.035 (4)	0.037 (3)	−0.012 (3)	−0.009 (3)	−0.013 (3)
C67	0.020 (3)	0.038 (4)	0.033 (3)	−0.008 (3)	−0.001 (2)	−0.011 (3)
C68	0.022 (3)	0.037 (4)	0.059 (4)	0.005 (3)	−0.008 (3)	−0.024 (3)
C69	0.031 (3)	0.034 (4)	0.055 (4)	−0.004 (3)	−0.008 (3)	−0.025 (3)
C70	0.021 (3)	0.023 (3)	0.013 (2)	−0.008 (2)	0.0004 (19)	−0.003 (2)
C71	0.045 (4)	0.016 (3)	0.019 (3)	−0.005 (3)	0.003 (2)	−0.001 (2)
C72	0.057 (4)	0.026 (4)	0.030 (3)	−0.004 (3)	0.012 (3)	−0.011 (3)
C73	0.054 (4)	0.037 (4)	0.015 (3)	−0.020 (3)	0.009 (3)	−0.007 (3)
C74	0.051 (4)	0.032 (4)	0.022 (3)	−0.008 (3)	0.004 (3)	−0.001 (3)
C75	0.033 (3)	0.027 (4)	0.022 (3)	0.004 (3)	0.006 (2)	0.000 (3)

### Geometric parameters (Å, °)

**Table:**

Sn1—C1	2.131 (6)	C30—C35	1.389 (7)
Sn1—C7	2.133 (6)	C31—C32	1.382 (7)
Sn1—C13	2.138 (5)	C31—H31	0.9500
Sn1—O1	2.239 (3)	C32—C33	1.382 (9)
Sn1—O3	2.221 (3)	C32—H32	0.9500
Ag1—P1	2.515 (1)	C33—C34	1.363 (9)
Ag1—P2	2.494 (1)	C33—H33	0.9500
Ag1—P3	2.495 (2)	C34—C35	1.391 (7)
Ag1—P4	2.518 (1)	C34—H34	0.9500
Cl1—C4	1.740 (5)	C35—H35	0.9500
Cl2—C20	1.785 (5)	C36—C37	1.539 (7)
Cl3—C22	1.728 (6)	C36—H36A	0.9900
F1—C20	1.345 (6)	C36—H36B	0.9900
F2—C20	1.322 (6)	C37—H37A	0.9900
F3—C22	1.343 (6)	C37—H37B	0.9900
F4—C22	1.363 (6)	C38—C43	1.392 (7)
Cl2'—C20	1.728 (7)	C38—C39	1.394 (7)
Cl3'—C22	1.704 (7)	C39—C40	1.392 (7)
F1'—C20	1.343 (8)	C39—H39	0.9500
F2'—C20	1.349 (8)	C40—C41	1.381 (8)
F3'—C22	1.393 (8)	C40—H40	0.9500
F4'—C22	1.345 (8)	C41—C42	1.369 (8)
P1—C23	1.819 (5)	C41—H41	0.9500
P1—C30	1.825 (5)	C42—C43	1.381 (7)
P1—C36	1.851 (5)	C42—H42	0.9500
P2—C44	1.823 (5)	C43—H43	0.9500
P2—C37	1.828 (5)	C44—C49	1.387 (7)
P2—C38	1.829 (5)	C44—C45	1.403 (7)
P3—C56	1.824 (5)	C45—C46	1.375 (7)
P3—C50	1.839 (5)	C45—H45	0.9500
P3—C62	1.841 (5)	C46—C47	1.389 (8)
P4—C70	1.829 (5)	C46—H46	0.9500
P4—C64	1.832 (5)	C47—C48	1.380 (8)
P4—C63	1.838 (5)	C47—H47	0.9500
O1—C19	1.286 (6)	C48—C49	1.390 (7)
O2—C19	1.221 (6)	C48—H48	0.9500
O3—C21	1.267 (7)	C49—H49	0.9500
O4—C21	1.224 (7)	C50—C55	1.387 (8)
C1—C6	1.394 (7)	C50—C51	1.390 (7)
C1—C2	1.395 (7)	C51—C52	1.384 (7)
C2—C3	1.393 (8)	C51—H51	0.9500
C2—H2	0.9500	C52—C53	1.395 (9)
C3—C4	1.386 (8)	C52—H52	0.9500
C3—H3	0.9500	C53—C54	1.357 (9)
C4—C5	1.382 (8)	C53—H53	0.9500
C4—H4	0.9500	C54—C55	1.401 (7)
C5—C6	1.379 (8)	C54—H54	0.9500
C5—H5	0.9500	C55—H55	0.9500
C6—H6	0.9500	C56—C57	1.390 (7)
C7—C8	1.392 (8)	C56—C61	1.416 (7)
C7—C12	1.400 (8)	C57—C58	1.379 (8)
C8—C9	1.373 (8)	C57—H57	0.9500
C8—H8	0.9500	C58—C59	1.393 (8)
C9—C10	1.388 (10)	C58—H58	0.9500
C9—H9	0.9500	C59—C60	1.368 (8)
C10—C11	1.359 (9)	C59—H59	0.9500
C10—Cl1'	1.718 (10)	C60—C61	1.390 (7)
C10—H10	0.9500	C60—H60	0.9500
C11—C12	1.389 (8)	C61—H61	0.9500
C11—H11	0.9500	C62—C63	1.526 (7)
C12—H12	0.9500	C62—H62A	0.9900
C13—C14	1.384 (7)	C62—H62B	0.9900
C13—C18	1.397 (7)	C63—H63A	0.9900
C14—C15	1.390 (8)	C63—H63B	0.9900
C14—H14	0.9500	C64—C65	1.376 (8)
C15—C16	1.386 (8)	C64—C69	1.376 (8)
C15—H15	0.9500	C65—C66	1.389 (8)
C16—C17	1.380 (8)	C65—H65	0.9500
C16—H16	0.9500	C66—C67	1.365 (8)
C17—C18	1.397 (8)	C66—H66	0.9500
C17—H17	0.9500	C67—C68	1.366 (9)
C18—H18	0.9500	C67—H67	0.9500
C19—C20	1.523 (6)	C68—C69	1.398 (8)
C21—C22	1.534 (6)	C68—H68	0.9500
C23—C24	1.397 (7)	C69—H69	0.9500
C23—C29	1.397 (8)	C70—C75	1.379 (8)
C24—C25	1.394 (8)	C70—C71	1.380 (7)
C24—H24	0.9500	C71—C72	1.387 (7)
C25—C27	1.379 (10)	C71—H71	0.9500
C25—H25	0.9500	C72—C73	1.366 (8)
C27—C28	1.373 (9)	C72—H72	0.9500
C27—H27	0.9500	C73—C74	1.385 (9)
C28—C29	1.386 (8)	C73—H73	0.9500
C28—H28	0.9500	C74—C75	1.380 (7)
C29—H29	0.9500	C74—H74	0.9500
C30—C31	1.386 (7)	C75—H75	0.9500
			
C1—Sn1—C7	127.5 (2)	C31—C30—P1	119.6 (4)
C1—Sn1—C13	112.4 (2)	C35—C30—P1	121.4 (4)
C1—Sn1—O3	91.1 (2)	C32—C31—C30	120.6 (5)
C1—Sn1—O1	85.5 (2)	C32—C31—H31	119.7
C7—Sn1—O1	91.8 (2)	C30—C31—H31	119.7
C7—Sn1—C13	120.1 (2)	C33—C32—C31	119.7 (6)
C7—Sn1—O3	89.9 (2)	C33—C32—H32	120.1
C13—Sn1—O3	87.2 (2)	C31—C32—H32	120.1
C13—Sn1—O1	94.5 (2)	C34—C33—C32	120.5 (6)
O1—Sn1—O3	176.6 (1)	C34—C33—H33	119.8
P1—Ag1—P2	82.98 (4)	C32—C33—H33	119.8
P1—Ag1—P3	132.80 (5)	C33—C34—C35	120.1 (6)
P1—Ag1—P4	122.85 (4)	C33—C34—H34	119.9
P2—Ag1—P3	119.12 (4)	C35—C34—H34	119.9
P2—Ag1—P4	119.90 (4)	C30—C35—C34	120.1 (6)
P3—Ag1—P4	83.87 (5)	C30—C35—H35	119.9
C23—P1—C30	103.0 (2)	C34—C35—H35	119.9
C23—P1—C36	103.0 (2)	C37—C36—P1	115.0 (4)
C30—P1—C36	101.9 (2)	C37—C36—H36A	108.5
C23—P1—Ag1	120.14 (17)	P1—C36—H36A	108.5
C30—P1—Ag1	121.86 (18)	C37—C36—H36B	108.5
C36—P1—Ag1	103.86 (16)	P1—C36—H36B	108.5
C44—P2—C37	105.6 (2)	H36A—C36—H36B	107.5
C44—P2—C38	102.5 (2)	C36—C37—P2	109.8 (3)
C37—P2—C38	103.0 (2)	C36—C37—H37A	109.7
C44—P2—Ag1	115.96 (17)	P2—C37—H37A	109.7
C37—P2—Ag1	100.48 (16)	C36—C37—H37B	109.7
C38—P2—Ag1	126.80 (18)	P2—C37—H37B	109.7
C56—P3—C50	104.1 (2)	H37A—C37—H37B	108.2
C56—P3—C62	103.9 (2)	C43—C38—C39	118.7 (5)
C50—P3—C62	103.3 (2)	C43—C38—P2	122.2 (4)
C56—P3—Ag1	125.32 (17)	C39—C38—P2	119.0 (4)
C50—P3—Ag1	113.64 (18)	C40—C39—C38	120.3 (5)
C62—P3—Ag1	104.17 (18)	C40—C39—H39	119.8
C70—P4—C64	100.8 (2)	C38—C39—H39	119.8
C70—P4—C63	103.5 (3)	C41—C40—C39	119.4 (6)
C64—P4—C63	104.2 (2)	C41—C40—H40	120.3
C70—P4—Ag1	113.72 (17)	C39—C40—H40	120.3
C64—P4—Ag1	129.67 (18)	C42—C41—C40	121.0 (5)
C63—P4—Ag1	102.04 (17)	C42—C41—H41	119.5
C19—O1—Sn1	118.7 (3)	C40—C41—H41	119.5
C21—O3—Sn1	121.1 (3)	C41—C42—C43	119.7 (6)
C6—C1—C2	117.9 (5)	C41—C42—H42	120.1
C6—C1—Sn1	117.7 (4)	C43—C42—H42	120.1
C2—C1—Sn1	123.9 (4)	C42—C43—C38	120.8 (5)
C3—C2—C1	121.1 (5)	C42—C43—H43	119.6
C3—C2—H2	119.4	C38—C43—H43	119.6
C1—C2—H2	119.4	C49—C44—C45	119.1 (5)
C4—C3—C2	119.0 (5)	C49—C44—P2	117.4 (4)
C4—C3—H3	120.5	C45—C44—P2	123.3 (4)
C2—C3—H3	120.5	C46—C45—C44	120.2 (5)
C5—C4—C3	121.0 (5)	C46—C45—H45	119.9
C5—C4—Cl1	118.4 (5)	C44—C45—H45	119.9
C3—C4—Cl1	120.6 (4)	C45—C46—C47	120.4 (5)
C5—C4—H4	119.5	C45—C46—H46	119.8
C3—C4—H4	119.5	C47—C46—H46	119.8
C6—C5—C4	119.2 (6)	C48—C47—C46	119.8 (5)
C6—C5—H5	120.4	C48—C47—H47	120.1
C4—C5—H5	120.4	C46—C47—H47	120.1
C5—C6—C1	121.7 (6)	C47—C48—C49	120.2 (6)
C5—C6—H6	119.1	C47—C48—H48	119.9
C1—C6—H6	119.1	C49—C48—H48	119.9
C8—C7—C12	118.3 (5)	C44—C49—C48	120.3 (5)
C8—C7—Sn1	122.9 (4)	C44—C49—H49	119.9
C12—C7—Sn1	118.6 (4)	C48—C49—H49	119.9
C9—C8—C7	121.1 (6)	C55—C50—C51	119.9 (5)
C9—C8—H8	119.4	C55—C50—P3	121.7 (4)
C7—C8—H8	119.4	C51—C50—P3	118.3 (4)
C8—C9—C10	119.1 (6)	C52—C51—C50	119.8 (5)
C8—C9—H9	120.4	C52—C51—H51	120.1
C10—C9—H9	120.4	C50—C51—H51	120.1
C11—C10—C9	121.4 (6)	C51—C52—C53	120.5 (6)
C11—C10—Cl1'	119.6 (11)	C51—C52—H52	119.7
C9—C10—Cl1'	118.9 (11)	C53—C52—H52	119.7
C11—C10—H10	119.3	C54—C53—C52	119.1 (5)
C9—C10—H10	119.3	C54—C53—H53	120.4
C10—C11—C12	119.5 (6)	C52—C53—H53	120.4
C10—C11—H11	120.3	C53—C54—C55	121.6 (6)
C12—C11—H11	120.3	C53—C54—H54	119.2
C11—C12—C7	120.5 (6)	C55—C54—H54	119.2
C11—C12—H12	119.8	C50—C55—C54	118.9 (6)
C7—C12—H12	119.8	C50—C55—H55	120.6
C14—C13—C18	117.6 (5)	C54—C55—H55	120.6
C14—C13—Sn1	121.4 (4)	C57—C56—C61	118.8 (5)
C18—C13—Sn1	121.0 (4)	C57—C56—P3	123.6 (4)
C13—C14—C15	121.4 (5)	C61—C56—P3	117.5 (4)
C13—C14—H14	119.3	C58—C57—C56	121.1 (5)
C15—C14—H14	119.3	C58—C57—H57	119.5
C16—C15—C14	120.8 (6)	C56—C57—H57	119.5
C16—C15—H15	119.6	C57—C58—C59	119.7 (5)
C14—C15—H15	119.6	C57—C58—H58	120.1
C17—C16—C15	118.6 (6)	C59—C58—H58	120.1
C17—C16—H16	120.7	C60—C59—C58	120.0 (5)
C15—C16—H16	120.7	C60—C59—H59	120.0
C16—C17—C18	120.7 (5)	C58—C59—H59	120.0
C16—C17—H17	119.7	C59—C60—C61	121.2 (5)
C18—C17—H17	119.7	C59—C60—H60	119.4
C17—C18—C13	121.0 (5)	C61—C60—H60	119.4
C17—C18—H18	119.5	C60—C61—C56	119.1 (5)
C13—C18—H18	119.5	C60—C61—H61	120.4
O2—C19—O1	129.4 (5)	C56—C61—H61	120.4
O2—C19—C20	116.6 (5)	C63—C62—P3	111.8 (4)
O1—C19—C20	113.8 (5)	C63—C62—H62A	109.2
F2—C20—F1	108.5 (4)	P3—C62—H62A	109.2
F1'—C20—F2'	110.9 (7)	C63—C62—H62B	109.2
F2—C20—C19	111.7 (4)	P3—C62—H62B	109.2
F1'—C20—C19	113.9 (6)	H62A—C62—H62B	107.9
F1—C20—C19	112.8 (4)	C62—C63—P4	111.5 (4)
F2'—C20—C19	114.3 (6)	C62—C63—H63A	109.3
F1'—C20—Cl2'	108.2 (6)	P4—C63—H63A	109.3
F2'—C20—Cl2'	104.7 (6)	C62—C63—H63B	109.3
C19—C20—Cl2'	103.9 (4)	P4—C63—H63B	109.3
F2—C20—Cl2	108.3 (4)	H63A—C63—H63B	108.0
F1—C20—Cl2	107.1 (4)	C65—C64—C69	119.2 (5)
C19—C20—Cl2	108.4 (3)	C65—C64—P4	118.3 (4)
O4—C21—O3	129.7 (5)	C69—C64—P4	122.4 (5)
O4—C21—C22	116.7 (5)	C64—C65—C66	120.7 (6)
O3—C21—C22	113.6 (5)	C64—C65—H65	119.7
F3—C22—F4	106.3 (5)	C66—C65—H65	119.7
F4'—C22—F3'	106.6 (7)	C67—C66—C65	120.4 (6)
F3—C22—C21	113.8 (5)	C67—C66—H66	119.8
F4'—C22—C21	112.7 (6)	C65—C66—H66	119.8
F4—C22—C21	110.9 (5)	C66—C67—C68	119.0 (6)
F3'—C22—C21	112.7 (5)	C66—C67—H67	120.5
F4'—C22—Cl3'	109.7 (6)	C68—C67—H67	120.5
F3'—C22—Cl3'	106.3 (6)	C67—C68—C69	121.4 (6)
C21—C22—Cl3'	108.5 (4)	C67—C68—H68	119.3
F3—C22—Cl3	108.7 (4)	C69—C68—H68	119.3
F4—C22—Cl3	107.1 (4)	C64—C69—C68	119.2 (6)
C21—C22—Cl3	109.7 (4)	C64—C69—H69	120.4
C24—C23—C29	118.9 (5)	C68—C69—H69	120.4
C24—C23—P1	121.2 (4)	C75—C70—C71	119.1 (5)
C29—C23—P1	119.8 (4)	C75—C70—P4	122.3 (4)
C25—C24—C23	119.6 (6)	C71—C70—P4	118.5 (4)
C25—C24—H24	120.2	C70—C71—C72	120.2 (5)
C23—C24—H24	120.2	C70—C71—H71	119.9
C27—C25—C24	120.6 (6)	C72—C71—H71	119.9
C27—C25—H25	119.7	C73—C72—C71	120.3 (6)
C24—C25—H25	119.7	C73—C72—H72	119.9
C28—C27—C25	120.3 (6)	C71—C72—H72	119.9
C28—C27—H27	119.9	C72—C73—C74	120.0 (5)
C25—C27—H27	119.9	C72—C73—H73	120.0
C27—C28—C29	119.9 (6)	C74—C73—H73	120.0
C27—C28—H28	120.0	C75—C74—C73	119.6 (6)
C29—C28—H28	120.0	C75—C74—H74	120.2
C28—C29—C23	120.7 (6)	C73—C74—H74	120.2
C28—C29—H29	119.6	C70—C75—C74	120.7 (5)
C23—C29—H29	119.6	C70—C75—H75	119.6
C31—C30—C35	119.0 (5)	C74—C75—H75	119.6
			
P2—Ag1—P1—C23	123.5 (2)	Ag1—P1—C23—C24	103.9 (4)
P3—Ag1—P1—C23	−112.8 (2)	C30—P1—C23—C29	147.4 (5)
P4—Ag1—P1—C23	2.3 (2)	C36—P1—C23—C29	41.6 (5)
P2—Ag1—P1—C30	−104.56 (19)	Ag1—P1—C23—C29	−73.1 (5)
P3—Ag1—P1—C30	19.1 (2)	C29—C23—C24—C25	−2.3 (8)
P4—Ag1—P1—C30	134.22 (19)	P1—C23—C24—C25	−179.3 (4)
P2—Ag1—P1—C36	9.28 (17)	C23—C24—C25—C27	0.8 (9)
P3—Ag1—P1—C36	132.94 (17)	C24—C25—C27—C28	0.3 (10)
P4—Ag1—P1—C36	−111.93 (17)	C25—C27—C28—C29	0.2 (10)
P3—Ag1—P2—C44	−54.5 (2)	C27—C28—C29—C23	−1.7 (9)
P1—Ag1—P2—C44	81.1 (2)	C24—C23—C29—C28	2.7 (9)
P4—Ag1—P2—C44	−154.87 (19)	P1—C23—C29—C28	179.7 (5)
P3—Ag1—P2—C37	−167.73 (16)	C23—P1—C30—C31	134.0 (4)
P1—Ag1—P2—C37	−32.09 (17)	C36—P1—C30—C31	−119.4 (4)
P4—Ag1—P2—C37	91.94 (17)	Ag1—P1—C30—C31	−4.6 (5)
P3—Ag1—P2—C38	77.2 (2)	C23—P1—C30—C35	−48.7 (5)
P1—Ag1—P2—C38	−147.1 (2)	C36—P1—C30—C35	57.8 (5)
P4—Ag1—P2—C38	−23.1 (2)	Ag1—P1—C30—C35	172.6 (3)
P2—Ag1—P3—C56	112.5 (2)	C35—C30—C31—C32	1.5 (7)
P1—Ag1—P3—C56	3.5 (2)	P1—C30—C31—C32	178.8 (4)
P4—Ag1—P3—C56	−126.6 (2)	C30—C31—C32—C33	−1.0 (8)
P2—Ag1—P3—C50	−17.01 (19)	C31—C32—C33—C34	0.2 (9)
P1—Ag1—P3—C50	−125.98 (18)	C32—C33—C34—C35	0.1 (9)
P4—Ag1—P3—C50	103.92 (18)	C31—C30—C35—C34	−1.3 (8)
P2—Ag1—P3—C62	−128.67 (18)	P1—C30—C35—C34	−178.5 (4)
P1—Ag1—P3—C62	122.37 (18)	C33—C34—C35—C30	0.5 (8)
P4—Ag1—P3—C62	−7.73 (18)	C23—P1—C36—C37	−104.1 (4)
P2—Ag1—P4—C70	−146.3 (2)	C30—P1—C36—C37	149.3 (4)
P3—Ag1—P4—C70	93.6 (2)	Ag1—P1—C36—C37	21.9 (4)
P1—Ag1—P4—C70	−44.5 (2)	P1—C36—C37—P2	−53.8 (4)
P2—Ag1—P4—C64	−17.2 (2)	C44—P2—C37—C36	−65.0 (4)
P3—Ag1—P4—C64	−137.3 (2)	C38—P2—C37—C36	−172.2 (3)
P1—Ag1—P4—C64	84.6 (2)	Ag1—P2—C37—C36	55.9 (3)
P2—Ag1—P4—C63	102.96 (18)	C44—P2—C38—C43	−37.0 (5)
P3—Ag1—P4—C63	−17.22 (18)	C37—P2—C38—C43	72.5 (5)
P1—Ag1—P4—C63	−155.30 (17)	Ag1—P2—C38—C43	−173.6 (4)
C1—Sn1—O1—C19	−177.6 (4)	C44—P2—C38—C39	141.4 (4)
C7—Sn1—O1—C19	55.0 (4)	C37—P2—C38—C39	−109.1 (5)
C13—Sn1—O1—C19	−65.3 (4)	Ag1—P2—C38—C39	4.8 (5)
C1—Sn1—O3—C21	−70.4 (4)	C43—C38—C39—C40	0.1 (8)
C7—Sn1—O3—C21	57.1 (4)	P2—C38—C39—C40	−178.4 (4)
C13—Sn1—O3—C21	177.2 (4)	C38—C39—C40—C41	−0.5 (9)
C7—Sn1—C1—C6	−128.5 (4)	C39—C40—C41—C42	0.4 (9)
C13—Sn1—C1—C6	49.7 (5)	C40—C41—C42—C43	0.1 (9)
O3—Sn1—C1—C6	−37.8 (4)	C41—C42—C43—C38	−0.5 (9)
O1—Sn1—C1—C6	142.7 (4)	C39—C38—C43—C42	0.4 (8)
C7—Sn1—C1—C2	59.6 (5)	P2—C38—C43—C42	178.9 (4)
C13—Sn1—C1—C2	−122.3 (4)	C37—P2—C44—C49	153.7 (4)
O3—Sn1—C1—C2	150.3 (4)	C38—P2—C44—C49	−98.8 (4)
O1—Sn1—C1—C2	−29.3 (4)	Ag1—P2—C44—C49	43.5 (4)
C6—C1—C2—C3	−0.1 (8)	C37—P2—C44—C45	−32.1 (5)
Sn1—C1—C2—C3	171.8 (4)	C38—P2—C44—C45	75.4 (5)
C1—C2—C3—C4	−1.6 (9)	Ag1—P2—C44—C45	−142.3 (4)
C2—C3—C4—C5	2.9 (9)	C49—C44—C45—C46	3.1 (7)
C2—C3—C4—Cl1	−176.8 (4)	P2—C44—C45—C46	−171.1 (4)
C3—C4—C5—C6	−2.5 (10)	C44—C45—C46—C47	−0.3 (8)
Cl1—C4—C5—C6	177.3 (5)	C45—C46—C47—C48	−2.5 (8)
C4—C5—C6—C1	0.7 (10)	C46—C47—C48—C49	2.5 (8)
C2—C1—C6—C5	0.6 (9)	C45—C44—C49—C48	−3.0 (7)
Sn1—C1—C6—C5	−171.9 (5)	P2—C44—C49—C48	171.5 (4)
C1—Sn1—C7—C8	−52.3 (5)	C47—C48—C49—C44	0.2 (8)
C13—Sn1—C7—C8	129.6 (5)	C56—P3—C50—C55	20.3 (5)
O3—Sn1—C7—C8	−143.7 (5)	C62—P3—C50—C55	−88.0 (5)
O1—Sn1—C7—C8	33.4 (5)	Ag1—P3—C50—C55	159.8 (4)
C1—Sn1—C7—C12	132.2 (4)	C56—P3—C50—C51	−157.0 (4)
C13—Sn1—C7—C12	−45.8 (5)	C62—P3—C50—C51	94.7 (4)
O3—Sn1—C7—C12	40.9 (4)	Ag1—P3—C50—C51	−17.5 (5)
O1—Sn1—C7—C12	−142.0 (4)	C55—C50—C51—C52	−2.8 (8)
C12—C7—C8—C9	−0.9 (8)	P3—C50—C51—C52	174.6 (4)
Sn1—C7—C8—C9	−176.4 (4)	C50—C51—C52—C53	1.3 (9)
C7—C8—C9—C10	2.4 (9)	C51—C52—C53—C54	0.2 (9)
C8—C9—C10—C11	−3.1 (9)	C52—C53—C54—C55	−0.1 (10)
C8—C9—C10—Cl1'	179.7 (10)	C51—C50—C55—C54	2.8 (8)
C9—C10—C11—C12	2.3 (9)	P3—C50—C55—C54	−174.5 (4)
Cl1'—C10—C11—C12	179.5 (11)	C53—C54—C55—C50	−1.3 (9)
C10—C11—C12—C7	−0.8 (9)	C50—P3—C56—C57	−106.0 (5)
C8—C7—C12—C11	0.1 (8)	C62—P3—C56—C57	1.8 (5)
Sn1—C7—C12—C11	175.7 (4)	Ag1—P3—C56—C57	120.8 (4)
C1—Sn1—C13—C14	−104.0 (5)	C50—P3—C56—C61	78.2 (4)
C7—Sn1—C13—C14	74.3 (5)	C62—P3—C56—C61	−174.0 (4)
O3—Sn1—C13—C14	−14.0 (5)	Ag1—P3—C56—C61	−55.0 (4)
O1—Sn1—C13—C14	168.9 (5)	C61—C56—C57—C58	1.0 (8)
C1—Sn1—C13—C18	74.4 (5)	P3—C56—C57—C58	−174.7 (4)
C7—Sn1—C13—C18	−107.3 (4)	C56—C57—C58—C59	1.1 (8)
O3—Sn1—C13—C18	164.4 (4)	C57—C58—C59—C60	−3.0 (8)
O1—Sn1—C13—C18	−12.6 (4)	C58—C59—C60—C61	2.7 (8)
C18—C13—C14—C15	0.6 (9)	C59—C60—C61—C56	−0.6 (8)
Sn1—C13—C14—C15	179.1 (5)	C57—C56—C61—C60	−1.3 (7)
C13—C14—C15—C16	0.5 (10)	P3—C56—C61—C60	174.7 (4)
C14—C15—C16—C17	−1.2 (9)	C56—P3—C62—C63	171.6 (4)
C15—C16—C17—C18	0.7 (9)	C50—P3—C62—C63	−80.0 (4)
C16—C17—C18—C13	0.5 (9)	Ag1—P3—C62—C63	39.0 (4)
C14—C13—C18—C17	−1.1 (8)	P3—C62—C63—P4	−59.8 (4)
Sn1—C13—C18—C17	−179.6 (4)	C70—P4—C63—C62	−71.2 (4)
Sn1—O1—C19—O2	−1.4 (8)	C64—P4—C63—C62	−176.2 (4)
Sn1—O1—C19—C20	173.5 (3)	Ag1—P4—C63—C62	47.1 (4)
O2—C19—C20—F2	−151.0 (5)	C70—P4—C64—C65	83.6 (5)
O1—C19—C20—F2	33.5 (6)	C63—P4—C64—C65	−169.3 (4)
O2—C19—C20—F1'	−4.8 (9)	Ag1—P4—C64—C65	−50.1 (5)
O1—C19—C20—F1'	179.6 (8)	C70—P4—C64—C69	−92.7 (5)
O2—C19—C20—F1	−28.5 (7)	C63—P4—C64—C69	14.4 (5)
O1—C19—C20—F1	155.9 (5)	Ag1—P4—C64—C69	133.6 (4)
O2—C19—C20—F2'	124.1 (9)	C69—C64—C65—C66	3.7 (9)
O1—C19—C20—F2'	−51.5 (9)	P4—C64—C65—C66	−172.7 (4)
O2—C19—C20—Cl2'	−122.3 (6)	C64—C65—C66—C67	−1.7 (9)
O1—C19—C20—Cl2'	62.1 (6)	C65—C66—C67—C68	−1.2 (9)
O2—C19—C20—Cl2	89.9 (5)	C66—C67—C68—C69	2.1 (10)
O1—C19—C20—Cl2	−85.7 (5)	C65—C64—C69—C68	−2.8 (9)
Sn1—O3—C21—O4	4.4 (9)	P4—C64—C69—C68	173.5 (5)
Sn1—O3—C21—C22	−174.8 (3)	C67—C68—C69—C64	−0.1 (10)
O4—C21—C22—F3	−163.4 (5)	C64—P4—C70—C75	57.7 (6)
O3—C21—C22—F3	15.8 (7)	C63—P4—C70—C75	−49.9 (5)
O4—C21—C22—F4'	19.4 (9)	Ag1—P4—C70—C75	−159.8 (5)
O3—C21—C22—F4'	−161.3 (8)	C64—P4—C70—C71	−118.8 (5)
O4—C21—C22—F4	−43.6 (8)	C63—P4—C70—C71	133.6 (5)
O3—C21—C22—F4	135.6 (6)	Ag1—P4—C70—C71	23.8 (5)
O4—C21—C22—F3'	140.2 (8)	C75—C70—C71—C72	0.1 (9)
O3—C21—C22—F3'	−40.6 (9)	P4—C70—C71—C72	176.7 (5)
O4—C21—C22—Cl3'	−102.3 (6)	C70—C71—C72—C73	−0.1 (10)
O3—C21—C22—Cl3'	76.9 (6)	C71—C72—C73—C74	0.6 (11)
O4—C21—C22—Cl3	74.5 (6)	C72—C73—C74—C75	−1.1 (11)
O3—C21—C22—Cl3	−106.2 (5)	C71—C70—C75—C74	−0.5 (9)
C30—P1—C23—C24	−35.7 (5)	P4—C70—C75—C74	−177.0 (5)
C36—P1—C23—C24	−141.4 (4)	C73—C74—C75—C70	1.1 (10)
